# Relationship between Cognitive Impairment and Behavioural Disturbances in Alzheimer’s Disease Patients

**DOI:** 10.3233/BEN-2010-0275

**Published:** 2010-11-23

**Authors:** Laura Serra, Roberta Perri, Lucia Fadda, Alessandro Padovani, Sebastiano Lorusso, Carla Pettenati, Carlo Caltagirone, Giovanni A. Carlesimo

**Affiliations:** ^1^Neuroimaging LaboratoryFondazione IRCCS Santa LuciaRomeItaly; ^2^Clinical and Behavioural NeurologyFondazione IRCCS Santa LuciaRomeItaly; ^3^Department of NeuroscienceUniversity of Rome Tor VergataRomeItaly; ^4^Department of Medical and Surgical SciencesUnit of Neurology University of BresciaSpedali Civili di BresciaBresciaItaly; ^5^Infermi HospitalRiminiItaly; ^6^Centro Regionale AlzheimerUOC Neurologia Ospedale Rho-PassiranaRhoItaly

**Keywords:** Cognitive functions, MDB, NPI-10, ADAS-Cog, BPSD, Alzheimer disease

## Abstract

*Background and Aims:* Alzheimer’s disease (AD) is a neurodegenerative disorder in which the patients can exhibit some behavioural disturbances in addition to cognitive impairment. The aims of the present study were to investigate the relationship between severity and rate of decline of the cognitive and behavioural impairment in patient with AD.

*Methods:* 54 AD patients were assessed at baseline and after 12 months with the Mental Deterioration Battery (MDB), the Alzheimer’s Disease Assessment ScaleCognitive (ADASCog) and the Neuropsychiatric Inventory (NPI10).

*Results:* MDB was more accurate than ADASCog in the early diagnosis of AD. Conversely, ADASCog was more sensitive at revealing the progression of cognitive decline. Depression, Apathy and Anxiety are the most frequent and severe behavioural disturbances at baseline. At followup Delusions and Irritability increased significantly. Significant correlations were observed between severity of cognitive impairment and behavioural disorders both at baseline and in the progression rate passing from T0 to T12.

*Conclusions:* Severity and progression rate of behavioural and cognitive alterations in patients with AD are significantly associated.

## 1. Introduction

Alzheimer's disease (AD) is a neurodegenerative disorder characterized by progressive impairment of cognitive functions [39] that is usually defined by its core cognitive features. Nevertheless, there is agreement in the literature that at some point in their illness patients with AD also exhibit behavioural disturbances [32] that are particularly distressing for family members and contribute greatly to the need for caregiving services [38]. Different studies have estimated the prevalence of behavioural and psychological symptoms in dementia (BPSD) [28]; in fact, estimates range from 25% to 80% [31,34,36]. The most frequent BPSD described during the course of AD are agitation [36] apathy [34,47], depression [8,21,46], anxiety [41] and delusions [6,25]. However, disinhibition [49], hallucinations [7,25], aggression [23], wandering and disturbances in eating behaviour [9] have also been described.

ISSN 0953-4180/10/$27.50 © 2010 – IOS Press and the authors. All rights reserved

Particularly debated in the literature is the relationship between BPSD and cognitive decline. Some authors have reported that specific behavioural disorders, such as psychotic symptoms and depression, are related to severity of the cognitive deficit [15,26,52] and can also influence rate of cognitive decline [2,5,11,21, 22,48,51]. On the contrary, other authors did not find such a relationship and concluded that cognitive and behavioural disturbances are substantially independent in AD [17,24,46]. These contrasting findings could be due to differences among studies in the criteria adopted to recruit patients and in the instruments used to assess cognitive and behavioural disorders. However, they may also reflect some peculiarity in the relationship pattern between particular BPSD and specific aspects of the cognitive impairment in demented patients. In fact, studies investigating the relationship between a wide spectrum of BPSD and general indexes of cognitive impairment have generally found only a weak association [21,36,40]. Conversely, studies evaluating the relationship between the impairment of individual cognitive functions and particular BPSD have usually found more specific and robust associations [5,25,30, 47,52].

The present study was aimed at further investigating the relationship between severity and qualitative aspects of the cognitive and behavioural impairment and between the rate of cognitive and behavioural decline over time in patients with AD. For this purpose, a group of patients with AD were submitted to a 12-month longitudinal evaluation of both behavioural disturbances and cognitive impairment. The 10-item version of the Neuropsychiatric Inventory (NPI-10) [14] was used to assess behavioural symptoms. The Mental Deterioration Battery (MDB) [10] and the Alzheimer's Disease Assessment Scale-Cognitive (ADAS-Cog) [43] were used to evaluate cognitive deficits. The MDB is a neuropsychological battery devised to detect early cognitive decline and to assess the qualitative characteristics of cognitive deficits. In a previous study, the MDB demonstrated good sensitivity and specificity in the diagnostic screening of patients with suspected dementia [10]. However, there are no data regarding the ability of the MDB to rate the progression of cognitive decline in demented patients. The ADAS-Cog is the most widely used scale for assessing the progression rate of cognitive decline in patients with AD [27,50]. According to some authors, the ADAS-Cog can also be used as a screening test for dementia. However, sensitivity and specificity in differentiating patients with dementia from healthy controls have rarely been reported [19,44, 53]. For this reason, a secondary aim of this study was to compare the two neuropsychological scales for sensitivity and specificity in the early diagnosis and assessment of the progression rate of cognitive deterioration in patients with AD.

## 2. Methods

### *2.1. Subjects*

Fifty-four AD patients (36 females, 18 males; mean age 73.1 $\pm$ 5.8; mean years of education 5.9 $\pm$ 2.7) were recruited from the Alzheimer's Disease Units of four neurological and geriatric centres in Italy from May 2000 to December 2002. Patients were diagnosed with probable AD based on the NINCDS-ADRDA criteria [35]. To include only patients affected by mild to moderate dementia, at the time of recruitment patients should be at the first diagnosis of AD and the Mini-Mental State Examination (MMSE) [20,33] score had to be above 15 (range 15–26.7, mean 20.72 $\pm$ 3.33). Based on a clinical interview, patients with clinical history of major psychiatric disorders (e.g, major depression, psychosis, mania) were excluded from the study. None of the included patients were taking anticholinesterase drugs at the time of study entry. During the year of our observation, all patients were given an anti-cholinesterase drug for the treatment of AD. In particular, 11 patients were assuming donepezil at a dosage of 5 mg/die, 11 patients donepezil at a dosage of 10 mg/die, 1 patient rivastigmine at a dosage of 3 mg, 8 patients rivastigmine at a dosage of 6 mg and 2 patients galantamine at a dosage of 8 or 16 mg/die. Some of them were also assuming antidepressants and/or benzodiapezines or neuroleptics. Local ethical committee approval and written informed consent from participants were obtained before the study began.

### *2.2. Procedure*

All patients were submitted to a baseline (T 0) and a 12-month follow-up (T 12) cognitive and behavioural evaluation. The entire assessment took two days (i.e. on the first day they received the MDB and on the second day the ADAS-Cog and NPI-10) with an interval of no more than 7 days.

#### *2.2.1. Cognitive evaluation*

AD patients received both the MDB [10] and the ADAS-Cog [18,43]. The MDB includes 8 cognitive tests that assess long-term verbal episodic memory (Immediate and Delayed recall of a 15-word list), shortterm memory for visual abstract stimuli (Immediate Visual Memory), executive functions (Phonological Verbal Fluency), language abilities (Sentence Construction), reasoning (Raven's Coloured Progressive Matrices) and constructional praxis (Copy of Drawings and Copy of Drawings with Landmarks). For each MDB test, normative data collected in an Italian population are available for score adjustment based on age and education and normality cut-off scores ($\geqslant$ 95% of the lower tolerance limit of the normal population distribution) [10]. In the normative study, a score below the normality cut-off in two or more of the eight tests of the battery was considered as the boundary between normal and pathological performance, and 93% sensitivity and 98% specificity were reached in the correct classification of demented patients and normal controls [10]. To assess the sensitivity of the MDB in detecting the participants' cognitive decline over the one-year period of our observation, we calculated an MDB total score by summing the $z$ scores on the eight tests of the battery (means and standard deviations of the Italian standardisation sample were used for this purpose) [10].

ADAS-Cog [43] consists of 11 tests that assess different cognitive areas: memory (Immediate 10-word list recall, Recognition of a 12-word list, Remembering of Test Instructions), praxis (Constructional Praxis and Ideational Praxis), language (Naming Objects and Fingers, Communicative ability, Comprehension, and Word Finding difficulties in spontaneous speech) and Time and Place orientation. For each test, performance level calculated as the number of errors made by the subject. The sum of the errors, adjusted for the subject's education level, provides a total score ranging from 0 to 70. A total score between 0 and 13 indicates the absence of dementia, between 14 and 17 questionable dementia, to be confirmed after six months, and total scores between 18 and 70 indicate progressively more severe forms of dementia.

#### 2.2.2. Behavioural assessment

For the behavioural assessment, patients' caregivers were asked to complete the NPI-10 [14] This scale permits separately quantifying the presence and severity of the following psychiatric symptoms: Delusions, Hallucinations, Agitation/Aggression, Dysphoria/Depression, Anxiety, Euphoria, Apathy, Disinhibition, Irritability/Lability and Aberrant Motor Behaviour. The score range for each item is 0-12 (derived from the rating of both severity and frequency of the behavioural disorder, with 0 corresponding to absence of the behavioural symptom and 12 to maximum frequency and severity of the disorder). A total score is obtained by summing the scores for each item.

## 3. Results

### 3.1. Cognitive scales: diagnostic accuracy and rate of cognitive decline

Twenty-six (48%) and 40 (74%) of the patients with AD were correctly classified as having dementia by the ADAS-Cog (total score $\geqslant$ 18) and the MDB (pathological scores on 2 or more tests), respectively. The diagnostic accuracy of the ADAS-Cog was significantly lower than that of the MDB (chi-square = 7.64; df 1; $p = 0.005$).

A significant decrease in cognitive functioning over the year of our observation was detected in the total score on the ADAS-Cog (T0 = 17.84 $\pm$ 6.38;. T12 = 21.40 $\pm$ 9.97; F = 8.19, $p = 0.006$) but not in the total score on the MDB (T0 = $-11.42 \pm 9.43$; T12 = $-12.05 \pm 10.29$; F = 0.21, $p$ = n.s.).

These results confirm the good sensitivity of the MDB in diagnosing even mild forms of dementia and of the ADAS-Cog in indicating the progression of cognitive decline. Therefore, in the following analyses we used the MDB as a measure of severity of cognitive impairment at T0 and the change from T0 to T12 on the ADAS-Cog as a measure of the rate of cognitive decline.

### 3.2. Severity and progression of behavioural impairment

As shown in Fig. 1, Dysphoria/depression and Apathy, followed by Agitation/Aggression and Anxiety were the most frequent behavioural symptoms (panel a) and those with the highest average severity (panel b) in the overall sample of AD participants. As revealed by a significant difference between the mean NPI-10 total scores at T0 (mean 12.91 $\pm$ 8.61) and T12 (mean 16.83 $\pm$ 11.27), a global worsening of BPSD occurred in our AD group (F = 6.26 $p = 0.015$). After 12 months, frequency of occurrence and severity of Dysphoria/depression, Apathy, Agitation/Aggression and Anxiety remained substantially the same. Conversely, Delusions and Irritability/Lability increased significantly in terms of the number of patients affected (chi-square = 3.97 and 5.11, df 1, $p$ = 0.046 and 0.023, respectively) and mean severity (Z = 2.19 and 2.83, $p$ = 0.028 and 0.004, respectively). Finally, Aberrant Motor Behaviour, Disinhibition, Euphoria and Hallucinations showed a low frequency of occurrence and severity at both T0 and T12.

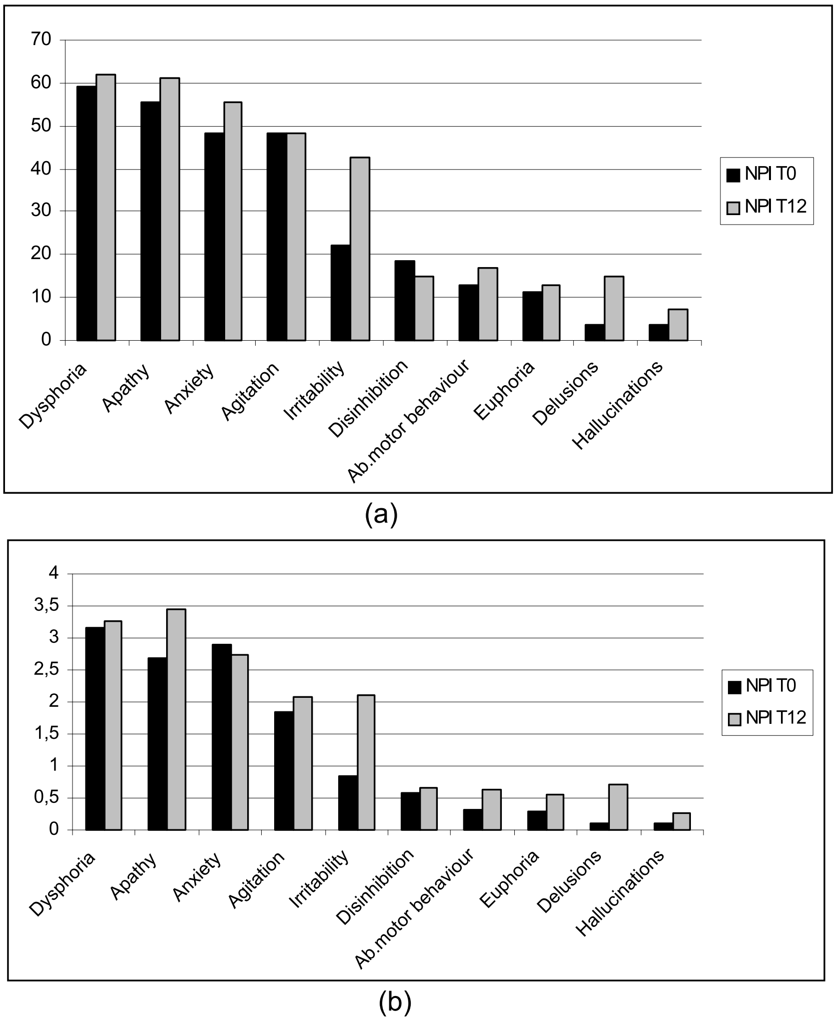

Fig. 1. Percentage of occurrence (panel A) and severity scores (panel B) for behavioural disturbances in the group of AD patients as indexed by NPI-10 subscales at T 0 and T 12.

Since some of AD patients included in the study were given an anticholinestease drug during the year between initial evaluation and follow-up, in a further analysis we wished to evaluate if the drug administration affected the rate of cognitive and/or behavioural decline in our sample of patients. For this purpose, we divided the overall sample in two subgroups, the first ($n = 31$) composed of individuals which were assuming an adequate dosage of anticholinestease drug (i.e., 6 or 12 mg/die of rivastigmine, 16 mg/die of galantamine and 5 mg or 10 mg/die of donepezil) and the second ($n = 23$) which included patients which were assuming no drug or a non adequate dosage (e.g., 3 mg/die of rivastigmine and 8 mg/die of galantamine). Two-way mixed ANOVAs with Group (adequate vs. no or non adequate treatment) as between factor and Time (T0 vs. T12) as within factor did not support a different rate of cognitive decline as a function of anticholinestease treatment. Indeed, the Group x Time interaction fell short of significance for both the total score on the MDB (F(1,52) $=$ 1.55, $p > 0.20$) and the total score on the ADAS-Cog (F(1,52) $=$ 0.40, $p > 0.20$). Quite unexpectedly, instead, patients who were assuming an adequate dosage of anticholinesterase drugs displayed an increment of NPI total scores larger than that disclosed by patients who were assuming no or non adequate dosage of therapy (F(1,52) $=$ 4.43, $p < 0.05$).

### 3.3. *Relationship between cognitive and behavioural impairment*

At T0, the overall severity score on the NPI-10 correlated significantly with the overall $z$ score on the MDB

($r = -0.38$; $p < 0.001$). Among the various MDB test scores, the Phonological Word Fluency test score was most consistently associated with the NPI-10 total score ($r = -0.46$; $p < 0.001$). Conversely, the severity score on the Disinhibition subscale of the NPI-10 had the most consistent relationship with the MDB total score ($r = -0.53$; $p < 0.001$). A stepwise forward multiple regression analysis with total MDB score as dependent variable and NPI-10 subscale scores as independent variables confirmed Disinhibition as the main behavioural predictor of cognitive performance at T0 in our AD population ($\beta = -0.53$; $t = 4.46$; $p < 0.001$).

Rate of cognitive decline (as measured by difference in ADAS-Cog score passing from T0 to T12) and progression rate of behavioural alterations (as measured by difference in NPI-10 total score passing from T0 to T12) were positively related ($r = 0.35$; $p < 0.001$). However, the total score on the NPI at T0 was not significantly correlated with the rate of cognitive decline measured by the ADAS-Cog ($r = 0.08$; $p =$ n.s.).

## 4. Discussion

The first result of this study is the demonstration that the two neuropsychological scales used, that is, the MDB and the ADAS-Cog, have different sensitivity in the early diagnosis and longitudinal assessment of cognitive decline in patients with AD. Indeed, confirming the ineffectiveness of the ADAS-Cog as a screening test for dementia [16], a significantly lower percentage of AD patients performed in the pathological range on the ADAS-Cog than on the MDB scale. One reason for the low sensitivity of the ADAS-Cog in detecting cognitive impairment may be the lack of a delayed recall condition in the verbal memory subtest. Indeed, consistent with the early localization of neuropathological changes in the mesio-temporal structures, which are critical for declarative memory functioning [3], delayed recall tests are much more sensitive than immediate recall tests in detecting memory impairment in patients with AD [12,45]. Indeed, the MDB correctly classified 74% of the patients with AD. Applying the same diagnostic criterion used here (i.e., two or more pathological scores on the eight tests comprising the battery), in a normative study [10] diagnostic sensitivity was 93% (with a specificity of 95% in identifying healthy controls). One possible reason for the lower diagnostic accuracy in the present than in the normative study is that the AD patients here were affected by mild to moderate forms of dementia (MMSE scores ranged from 15 to 25), whereas the AD sample used for the MDB standardization also included individuals with more severe forms of dementia.

The findings of the present study confirm the reliability of the ADAS-Cog in rating cognitive decline over time in patients with dementia [16]. In fact, the total score decrement passing from the baseline to the 12-month follow-up was significant for the ADAS-Cog but not for the MDB. The high sensitivity of the ADAS-Cog in revealing even mild changes in cognitive efficiency is likely due to the wide range of the total score (0–70), which permits graduating step by step the evolution of cognitive symptoms [43]. Conversely, the poor sensitivity of the MDB in registering the progressive decrement of cognitive efficiency is likely due to the fact that on some tests in the battery (i.e., delayed recall of the word list) AD patients reach a floor effect of performance precociously and thus fail to evidence further worsening at the follow-ups.

A second result of the present study is the demonstration that Dysphoria/Depression, Apathy and Anxiety are the most frequent and severe BPSD in patients with AD. These results are in substantial agreement with findings of previous studies that also used NPI-10 to investigate the prevalence and the qualitative features of BPSD in the early phases of AD [21,46]. Conversely, we found a particularly low prevalence of positive psychotic symptoms (both Delusions and Hallucinations) in our AD sample. This is at variance with results of previous studies reporting a prevalence of over 40% of Delusions in samples of patients with AD [21,25,26, 52]. The prevalence of hallucinations in AD has been estimated differently: Some studies have reported a prevalence of over 30–40% [29,52] and others less than 10% [25,26]. Only Spalletta et al. [46] reported a low occurrence of both Delusions and Hallucinations, similar to the findings of the present study. Discrepancies among studies regarding the prevalence and severity of reported BPSD are likely due to differences in the average severity of cognitive deterioration in the investigated samples and to sensitivity of the diagnostic protocols used to assess BPSD. In fact, the low prevalence of psychotic symptoms in our sample could be related to the particularly early phase of cognitive deterioration in our AD group (as demonstrated by an average MMSE score over 20) and to the use of a testing instrument (NPI-10) that assesses a wide range of BPSD and, therefore, could be less sensitive to the presence of psychotic symptoms than specifically devoted instruments (e.g., the ad hoc structured interviews used by Mizrahi et al. [37] and Wilson et al. [52].

In the literature, less attention has been paid to the longitudinal course of BPSD in AD patients. In particular, there is no clear consensus as to whether behavioural and psychological symptoms worsen homogeneously during the course of AD or, instead, whether a different progression over time characterises various behavioural symptoms [38]. At the 12-month follow-up, we documented an overall worsening of behavioural symptoms, as revealed by the increase in the total NPI-10 score. However, a detailed analysis of the subscales revealed that while most of the symptoms detected by NPI-10 remained substantially stable during the period of our observation (including Dysphoria/Depression, Apathy, Anxiety and Agitation/Aggression), both prevalence and average severity of Delusions and Irritability/Lability increased significantly. These results are in substantial agreement with the literature, which documents a progressive worsening of psychotic symptoms during the course of the disease [13,30].

Incidentally, we found that while the anticholinesterase therapy did not significantly affect the rate of cognitive decline (as indexed by total scores on both the ADAS-Cog and MDB) in our AD sample, patients who were assuming an adequate dosage of anticholinesterase drug presented a steeper worsening of behavioural symptoms (as measured by total NPI score) than patients who were assuming no therapy or a non adequate dosage of the drugs. We do not have a clear explanation for such unexpected data. In a previous systematic review of available literature data, Rodda et al. [42] reported the effect of anticholinesterase treatment over the behavioural disturbances in patients with AD did not differ from that of placebo. It is possible that in our AD sample the development of more severe behavioural disturbances was actually responsible of the earlier onset and more rapid dosage increase of anticholinesterase treatment in some of the recruited patients. Further studies are obviously needed to get more insight on this issue.

There is a general consensus in the literature that severity of cognitive decline and prevalence and gravity of behavioural symptoms are positively related in patients with AD. Such a relationship has been described most frequently between severity of individual BPSD and a general index of cognitive impairment, such as the MMSE score [21,25,47]. However, more specific associations between particular behavioural symptoms (such as Delusions or Apathy) and individual neuropsychological deficits have been reported as well [29,52]. In the present study, we found that at T0 the NPI-10 total score correlated significantly with performance on most of the MDB tests. A more specific relationship emerged between the severity score on the NPI-10. Disinhibition scale and performance level on most of the MDB tests and with the total MDB score. On the other side, performance scores on the Phonological Word Fluency test showed the most consistent relationship with individual NPI-10 scale scores. It is generally held that poor performance on a test of Phonological Word Fluency is the expression of frontal dysfunction [1]. On the other hand, factor analyses of severity scores on the various NPI-10 subscales have repeatedly demonstrated that Disinhibition correlates with the expression of other behavioural symptoms of frontal sufferance, e.g., Euphoria [21,46]. Taken together, these results suggest that neuropathological changes at the level of cortical frontal regions are responsible for the appearance of most behavioural symptoms in AD patients [4].

As further support of the parallelism between the temporal course of behavioural and cognitive symptoms in AD, we found that rate of cognitive decline passing from T0 to the T12 follow-up (as expressed by a change in the overall ADAS-cog score) and worsening of the behavioural symptoms during the same period (as manifested by an increase in the overall NPI-10 score) were significantly correlated. However, neither the overall NPI-10 score nor the partial scores on the specific NPI-10 subscales obtained at T0 were able to reliably predict the rate of cognitive decline. A similar failure of the BPDS to provide a prognostic index of the successive rate of cognitive deterioration has been reported by other authors. Nevertheless, Frisoni et al. [21] and Wilson et al. [52] reported that AD individuals with psychotic symptoms presented a steeper cognitive decline than individuals without psychosis. As noted above, in our AD sample prevalence and severity of psychotic symptoms (both Delusions and Hallucinations) were particularly low, possibly accounting for the lack of support to the above mentioned predictive role of these variables over the rate of cognitive decline.

In conclusion, the results of the present study highlight a substantial parallelism between cognitive and behavioural alterations in AD. Indeed, the severity of cognitive and behavioural symptoms at the time of study entry and the rate of decline during the year of our observation were significantly related. A more qualitative analysis of the pattern of correlation between MDB and NPI-10 sub-scores suggests an association between BPSD (particularly Disinhibition) and neuropsychological signs of frontal dysfunction. Finally, our results provide no indication that severity of BPSD in the early phases of AD is predictive of the rate of subsequent cognitive decline. One strength of the present study was our decision to use cognitive scales that demonstrated different sensitivity in detecting the initial symptoms of cognitive impairment (MDB) or in following the rate of cognitive decline over time (ADAS-Cog) in patients with AD. This allowed us to detect an association between behavioural and cognitive alterations in both the cross-sectional (T0) and the longitudinal (from T0 to T12) part of our study. Conversely, a limit of the study was the relatively small AD sample size and the use of a testing instrument, the NPI-10, that covers the whole spectrum of possible behavioural alterations and likely has little sensitivity for detecting mild alterations (e.g., in the domain of psychotic symptoms). Further studies on larger patient populations and more focused on specific symptoms are needed to gain more insight into the relevance of behavioural alterations and their relationship to cognitive deficits in AD.
